# Comprehensive Analyses of Mutation-Derived Long-Chain Noncoding RNA Signatures of Genome Instability in Kidney Renal Papillary Cell Carcinoma

**DOI:** 10.3389/fgene.2022.874673

**Published:** 2022-04-25

**Authors:** Jian Li, Shimei Wei, Yan Zhang, Shuangshuang Lu, Xiaoxu Zhang, Qiong Wang, Jiawei Yan, Sanju Yang, Liying Chen, Yunguang Liu, Zhijing Huang

**Affiliations:** ^1^ Department of Pediatrics, Affiliated Hospital of Youjiang Medical University for Nationalities, Baise, China; ^2^ Department of Pediatrics, Shanxi Children's Hospital, Taiyuan, China; ^3^ Graduate School of Youjiang Medical University for Nationalities, Baise, China

**Keywords:** genomic instability, KIRP, lncRNAs, mutator phenotype, prognostic

## Abstract

**Background:** The role of long-chain noncoding RNA (lncRNA) in genomic instability has been demonstrated to be increasingly importance. Therefore, in this study, lncRNAs associated with genomic instability were identified and kidney renal papillary cell carcinoma (KIRP)-associated predictive features were analysed to classify high-risk patients and improve individualised treatment.

**Methods:** The training (*n* = 142) and test (*n* = 144) sets were created using raw RNA-seq and patient’s clinical data of KIRP obtained from The Cancer Genome Atlas (TCGA).There are 27 long-chain noncoding RNAs (lncRNAs) that are connected with genomic instability, these lncRNAs were identified using the ‘limma’ R package based on the numbers of somatic mutations and lncRNA expression profiles acquired from KIRP TCGA cohort. Furthermore, Cox regression analysis was carried out to develop a genome instability-derived lncRNA-based gene signature (GILncSig), whose prognostic value was confirmed in the test cohort as well as across the entire KIRP TCGA dataset.

**Results:** A GILncSig derived from three lncRNAs (BOLA3-AS1, AC004870, and LINC00839), which were related with poor KIRP survival, was identified, which was split up into high- and low-risk groups. Additionally, the GILncSig was found to be an independent prognostic predictive index in KIRP using univariate and multivariate Cox analysis. Furthermore, the prognostic significance and characteristics of GilncSig were confirmed in the training test and TCGA sets. GilncSig also showed better predictive performance than other prognostic lncRNA features.

**Conclusion:** The function of lncRNAs in genomic instability and the genetic diversity of KIRP were elucidated in this work. Moreover, three lncRNAs were screened for prediction of the outcome of KIRP survival and novel insights into identifying cancer biomarkers related to genomic instability were discussed.

## Introduction

Kidney renal papillary cell carcinoma (KIRP) accounts for approximately 15% of all renal cell carcinoma (RCC) cases, which is the second most common type of RCC after clear cell RCC (Znaor et al., 2015). KIRP, a renal parenchymal malignancy, includes two subtypes (type 1 and type 2), with type 2 having a worse prognosis. The cause of KIRP remains unclear, the loss or mutation of the von Hippel Lindau (VHL) gene is observed in roughly 85% of KIRP tumours, which is one of the inevitable initial steps in the development of KIRP. It has been reported that VHL mutation through Akt/GSK-3 β Signalling pathway mediated SALL4 overexpression to promote the occurrence and vascularization of clear cell renal cell carcinoma (Sun J. et al., 2020). In addition, chromosome abnormality is another important factor in the occurrence and development of KIRP, such as Chromosome 3p Deletion (Jonasch et al., 2021) and Xp11.2 translocation (Dong et al., 2021). At present, clinicopathological features are considered the main diagnostic criteria for KIRP; however, the prediction results are inaccurate owing to the inconsistent criteria.(Sukov et al., 2012). Recently, various studies have focussed on developing potential targets that could benefit KIRP treatment, such as foretinib; however, these medications are only effective against type 1 KIRP and not to the aggressive type 2 KIRP (Choueiri et al., 2013; Choueiri et al., 2014). Currently, a standard treatment method for KIRP is not available, and the mortality of patients with KIRP is still very high. Therefore, identifying new biomarkers and formulating effective diagnosis and treatment strategies, which can improve the survival rate of patients with KIRP, is crucial.

Genomic instability induced by imperfect mismatch repair systems is characterised by extensive mutations throughout the genome, especially in highly repetitive microsatellite regions. This suggests that genomic instability can be used as a predictor of illness outcome, with the association of mutation accumulation to tumour advancement and survival (Suzuki et al., 2003; Ottini et al., 2006). Moreover, the underlying mechanism of genomic instability requires further elucidation. A study reports the potential use of molecular features to quantitatively measure genomic instability (Tam et al., 2019). For example, a study found 10 miRNA markers related to genomic instability that were associated with ovarian cancer (OV) prognosis (Zeng et al., 2018; Zhang et al., 2019). In the past decade, high-throughput genome sequencing technology has promoted the discovery of various prognostic factors, such as long-chain noncoding RNA (lncRNA). lncRNA is a common noncoding RNA that has a length of no less than 2000 nucleotides and is involved in the development of cancer [8]. Its abnormal expression or behaviour impacts cancer development (Chi et al., 2019). For example, lncRNA *Arlnc1* promotes androgen receptor-regulated prostate cancer progression (Zhang et al., 2018), and *Malat-1* promotes tumour epithelial–mesenchymal transformation (EMT) and further promotes the progression of RCC (Zhang et al., 2015). Additionally, numerous studies have published that lncRNAs are involved in cell survival, proliferation, migration and genomic stability (Moran et al., 2012; Iyer et al., 2015; Yao et al., 2019; Zhou et al., 2019). Some genomic instability-related lncRNA indicators have recently been found to predict prognosis in cancer patients, such as lung adenocarcinoma (Peng et al., 2021) and colon cancer (Yin et al., 2021). However, the potential biological process and genomic instability related to the clinical significance of lncRNA in KIRP remain unclear.

In this study, we used The Cancer Genome Atlas (TCGA) to develop a computational framework derived from the mutational hypothesis of lncRNA expression profile and somatic mutation profile in tumour genomes, which can be used as prognostic risk models, were identified to improve patient stratification and promote personalised treatment decision-making.

## Materials and Methods

### Data Collection

Clinical characteristics, RNA-seq and somatic mutation data of patients with KIRP were downloaded from TCGA database, including the expression profiles of paired lncRNA and mRNA, somatic mutations, patient’s survival information and clinicopathological characteristics. The training set comprised TCGA dataset, which included 321 patients who were divided at random into the training (144 patients) and test groups (142 patients). These groups were carried out in order to find lncRNA signatures that could be used for prognostic index for KIRP and construct prognostic risk models. Additionally, another independent KIRP cancer validation set (GSE3494) was downloaded from the GEO database, consisting of a large number of participants and Corresponding clinicopathological characteristics.

### Screening of lncRANs Related to Genomic Instability

Somatic mutation information and matched lncRNA expression profiles were obtained from the KIRP-TCGA dataset in order to identify lncRNAs that are associated with genomic instability. The total number of somatic mutations in each patient was calculated using Perl. The subjects were sorted in descending order according to the number of somatic mutations. The genomic unstable (GU) group consisted of the top 25% of samples with the greatest mutation frequency, while the bottom 25% of the samples with the lowest mutation frequency was considered as the genomic stable (GS) group. The expression patterns of these two groups were compared to identify lncRNAs that are linked to genomic instability using Wilcoxon test analysis with the package of ‘limma’. *p* < 0.05 and |log2 (FC)| > 1 were determined to the screening thresholds.

### Analyses of Hierarchical Clustering and Construction of a Co-expression Network

Depending on the levels of expression of the selected genome instability-related lncRNAs, a hierarchical clustering analysis was done using ‘limma’ (Liu et al., 2021) and ‘sparcl’ (Yin et al., 2021) packages to separate the patients into two groups: GU-like (GU^like^) and GS-like (GS^like^). The top 10 mRNAs that were highly linked with each lncRNA were chosen as target genes using the Pearson correlation analysis to construct the lncRNA and mRNA co-expression network.

### Establishment of a Prognostic Signature

Survival analysis was used to assess the role of genomic instability-associated lncRNAs prognostic using the ‘survival’ R package. Further, univariate and multivariate Cox regression analyses were performed to investigate the relationship between lncRNA expression and clinical prognosis. Based on the expression levels of the genome instability-related prognostic lncRNAs and their associated coefficient, the genomic instability-associated lncRNA prognostic index was constructed as follows:

GILncSig(riskscore)=∑=1ncoef(lncRNAi)∗expr(lncRNAi)

GILncSig is a prognostic risk score for patients with KIRP. KIRP patients were divided into the high-risk (risk^hi^) and low-risk (risk^lo^) groups in accordance with the median value of GILncSig. The Kaplan–Meier method was carried out to calculate the prognostic value of the GILncSig based on the middle of GILncSig scores, and multivariate Cox regression and hierarchical analyses were performed to determine the independence of GILncSig against other key clinicopathological features. Additionally, the receiver operating characteristic (ROC) curve (AUC) was performed to examine the prediction accuracy of GILncSig.

### Functional and Pathway Enrichment Analyses

The enrichment analysis of the Gene Ontology (GO) and Kyoto Encyclopedia of Genes and Genomes (KEGG) were carried out on the mRNA in the co-expression network using the ‘clusterProfiler’ package (Yu et al., 2012) with the threshold of *p* < 0.05.

## Results

### Identification of Genomic Instability-Associated lncRNAs in KIRP Patients

**FIGURE 1:**
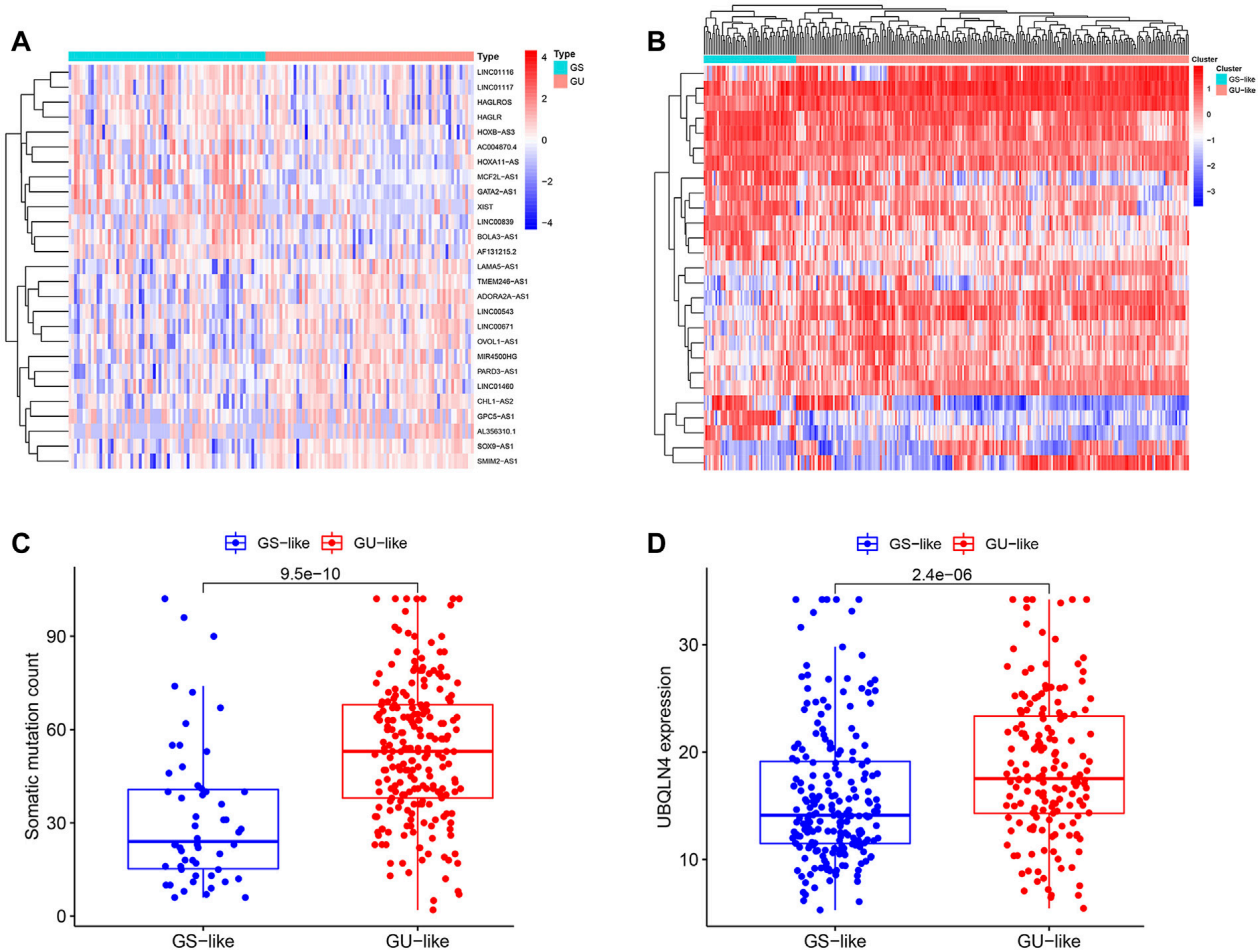
Identification of genomic instability-associated lncRNAs in patients with KIRP. **(A)**. Unsupervised clustering of 321 patients with KIRP was performed according to the quantification of the expression of 27 lncRNAs. The blue cluster on the left indicates the GS^like^ cluster, while the red cluster on the right indicates the GU^like^ cluster. **(B)**. Heat maps of 27 genomic instability-related lncRNAs in the GS^like^ and GU^like^ clusters. **(C)**. Boxplots of somatic mutation counts in the GS^like^ and GU^like^ clusters. **(D)**. Comparison of UBQLN4 expression level between the GS^like^ and GU^like^ clusters.

**FIGURE 2:**
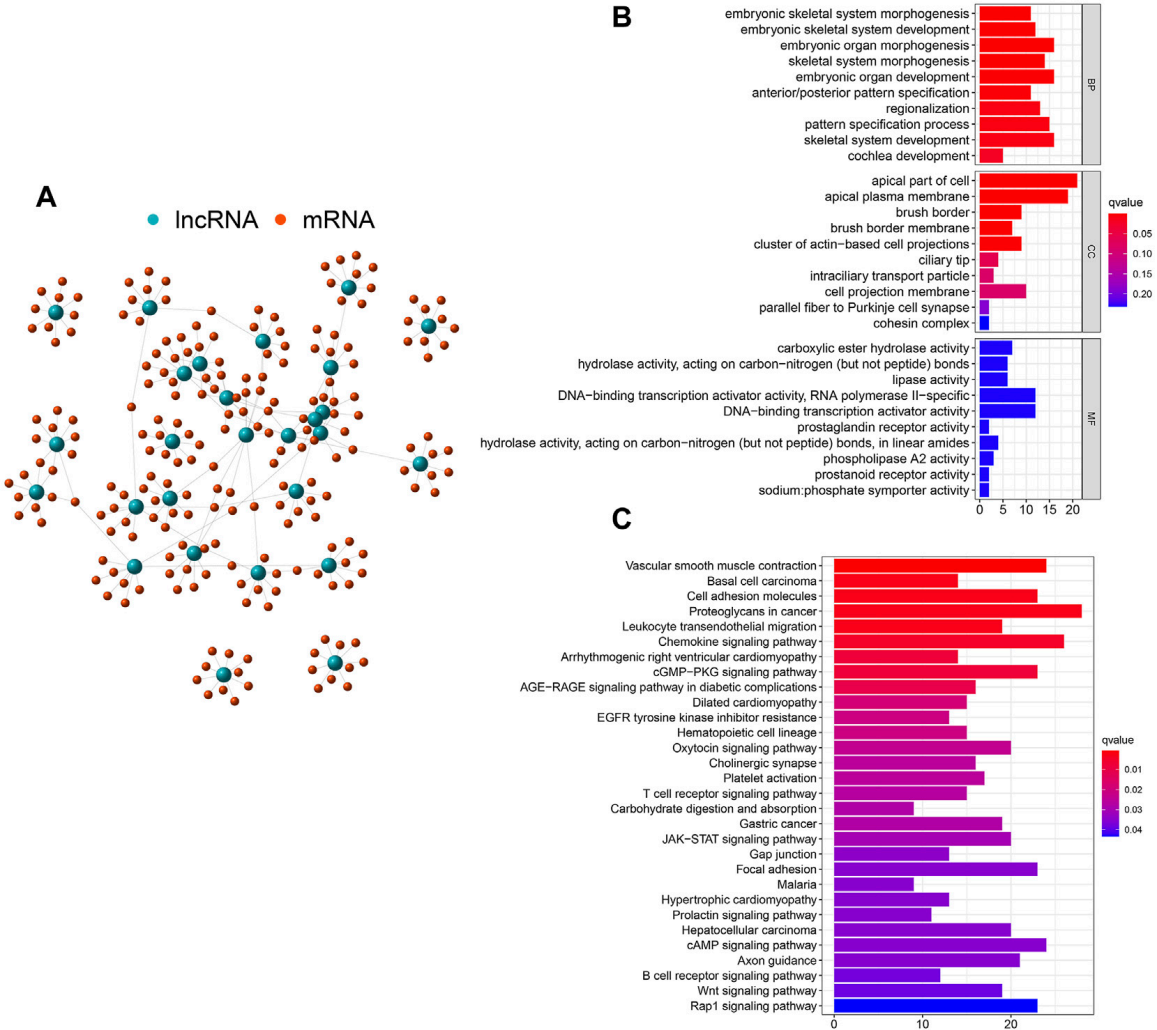
Functional annotations of genomic instability-related lncRNAS in patients with KIRP. **(A)**.Co-expression network of mRNAs and lncRNAs associated with genomic instability. Gene Ontology **(B)** and Kyoto Encyclopedia of Genes and Genomes enrichment analysis **(C)** of the lncRNA co-expressed mRNAs.

Based on the cumulative somatic mutation count per patient, the top 25% (*n* = 61) and the bottom 25% (*n* = 61) of the KIRP subjects were assigned to the GU^like^ and GS^like^ groups. A total of 27 differentially expressed lncRNAs were determined at *p* < 0.05 and |log2 (FC)| > 1 by comparing differences in lncRNA expression, which revealed 14 up-regulated and 13 down-regulated lncRNAs in the GU^like^ group (Supplementary Table S1). These lncRNAs that were up-regulated and down-regulated were chosen to visualize a heat map (Figure 1A). The expression level of 155 genomic instability-related lncRNAs was used to divide the subjects into the GU^like^ or GS^like^ clusters (Figure 1B). Moreover, compared with GS^like^ clusters, the GU^like^ cluster had a higher frequency of somatic mutations (Figure 1C). Besides, the UBQLN4 expression in GU^like^ cluster group is remarkably higher than in the GS^like^ clusters (Figure 1D). The biological functions of 146 lncRNAs related target genes were analysed using the GO and KEGG pathway enrichment analyses. The target genes were chosen from the top 10 mRNAs that were highly associated with each lncRNA. Subsequently, the lncRNA and mRNA co-expression network was built based on the correlation between the lncRNAs and mRNAs (Figure 2A). GO analysis revealed that (Figure 2B) the biological processes of the identified mRNAs were mainly enriched in embryonic organ morphogenesis, embryonic organ development and skeletal system development, with the cell components mainly concentrated in the apical cell, apical plasma membrane and brush border. The molecular functions were mainly concentrated during skeletal system development, gastric acid secretion regulation and proximal/distal pattern formation. KEGG pathway analysis of the most identified target genes was concentrated in proteoglycans for cancer, chemokine signalling pathway and cAMP signalling pathway (Figure 2C).

### Development of GILncSig as a Prognostic Index in the Training Set

**FIGURE 3:**
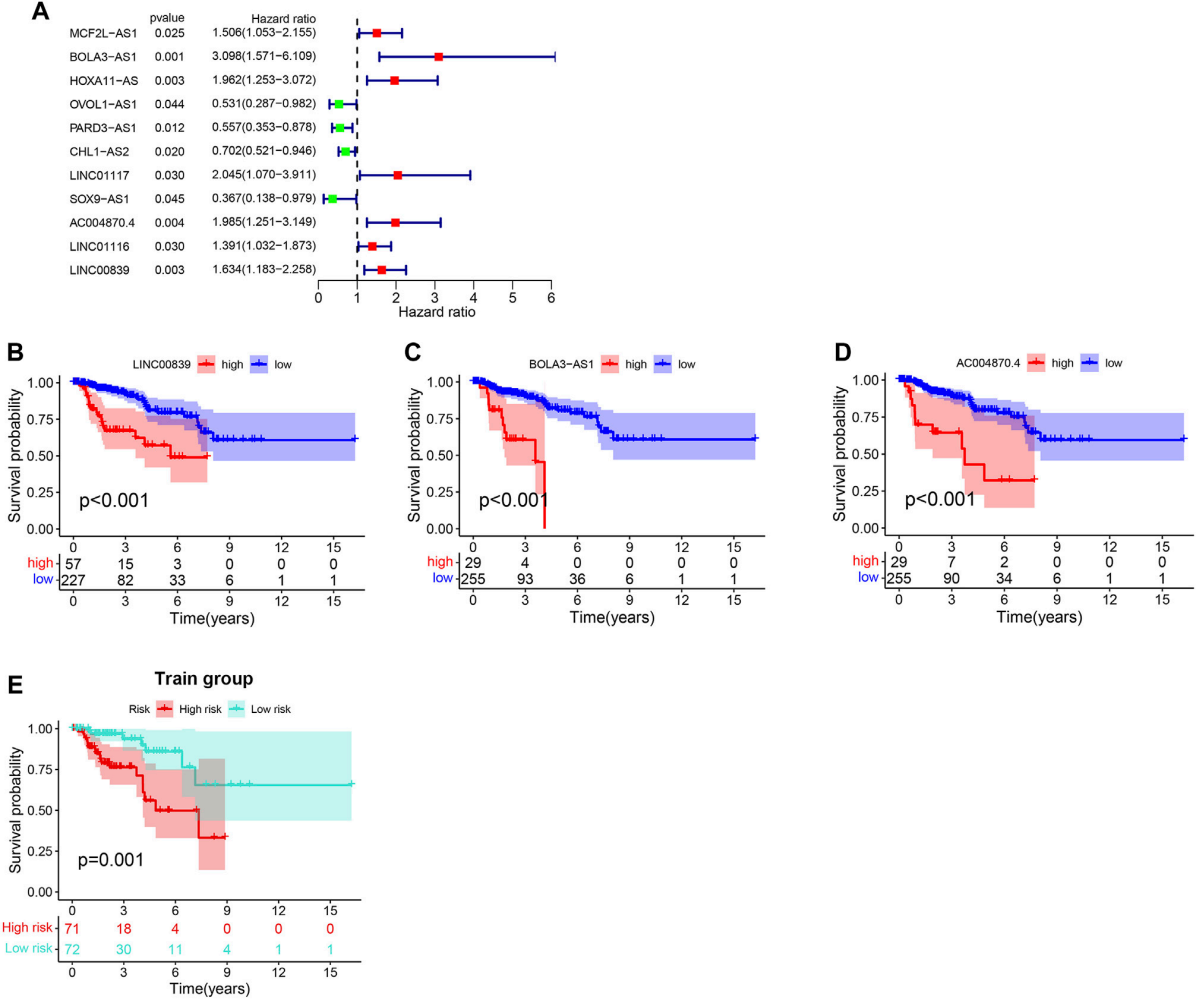
Construction of GILncSig for outcome prediction in the training set. **(A)**. Forest map of the genomic instability-related lncRNAs associated with survival. **(B–D)**.Kaplan-Meier curves from 27 genomic instability-related lncRNAs in KIRP patients. **(E)**.Kaplan–Meier curves for distinct risk groups stratified based on GILncSig in the training set.

**FIGURE 4:**
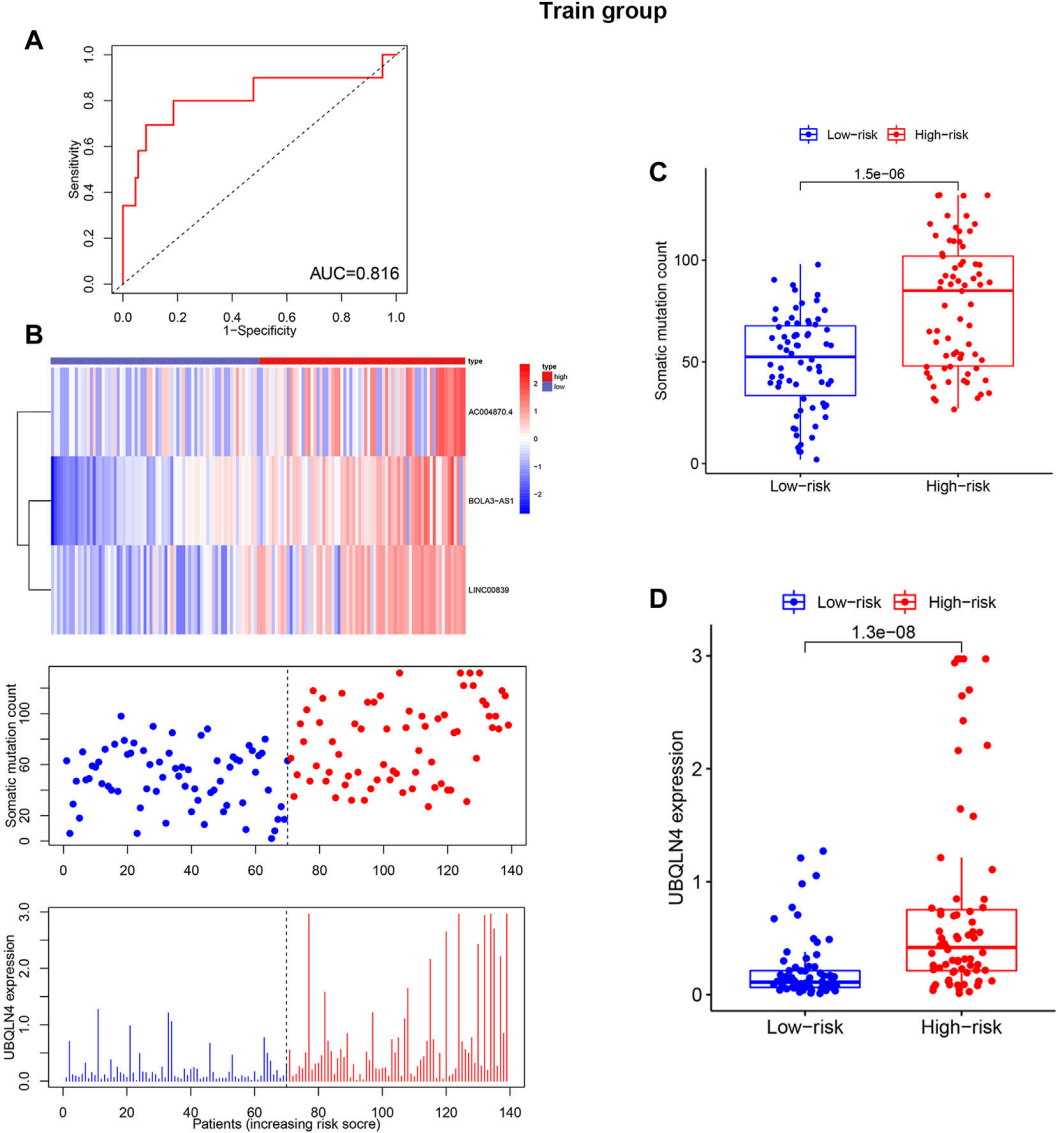
Independent validation of GILncSig in the training set with RNA-seq platform. **(A)**. Time receiver operating characteristic curve analysis of GILncSig in the training set at 3 years. **(B)**. lncRNA expression patterns, somatic mutation distribution and UBQLN4 expression based on the increasing trend of GILncSig score. Additionally, total somatic mutation distribution with increasing GILncSig score is displayed. **(C)**. Comparison of the total number of somatic mutations in different GILncSig groups. **(D)**. The expression level of UBQLN4 in various GILncSig groups.

The training (*n* = 144) and test sets (*n* = 142) were created by randomly dividing all samples received from the TCGA database. In the training set, 14 prognoses-related lncRNAs were screened from 27 genomic instability-related lncRNAs using Univariate Cox analysis, and a forest plot was drawn (Figure 3A). Additionally, the survival curve also revealed that the selected genes were linked to a poor prognosis in patients with KIRP, except ADORA2A-AS1, GPC5-AS1, OVOL1-AS1, RARD3-AS1, CHL1-AS2, and SOX9-AS1 (Supplementary Figure S1, Figure 3B). The univariate and multivariate Cox proportional hazards regression analysis revealed three lncRNAs that were identified as high risk factors (HR > 1) and independent prognostic lncRNAs (Supplementary Table S2, Figures 3A–D). A GILncSig was created to assess the prognostic risk of individuals with KIRP based on the expression level of the three independent prognostic genomic instability-associated lncRNAs using multivariate Cox analysis as follows: LINC00839 expression level × 0.339 + BOLA3-AS1 expression level × 0.734 AC004870 expression level × −0.687. It was consistent with the previous analysis results, of the GILncSig, the coefficient of these three lncRNAs was positive, indicating that these lncRNAs may be a risky factor because their high expression was associated with a poor prognosis. When the median risk score for the training concentration was utilized as the dividing point, we found that the KIRP patients with risk high group had worse survival rate (Figure 3E). The AUC of GILncSig was 0.816 according to the ROC curve (Figure 4A), which further confirmed its role as a prognostic marker of KIRP. Furthermore, the patients were ranked in ascending order according to their risk score for observing GILncSig trends, somatic mutation numbers and UBQLN4 expression value. Patients with risk^hi^ scores showed an increasing trend in somatic mutation count and UBQLN4 expression level (Figure 4B). Moreover, compared with the risk^lo^ group, the high-risk group had more numbers of somatic mutations (Figure 4C). Similarly, the risk^hi^ group showed higher expression levels of UBQLN4 than the risk^lo^ group (Figure 4D).

### Validation of the GILncSig in a Distinct Dataset

**FIGURE 5:**
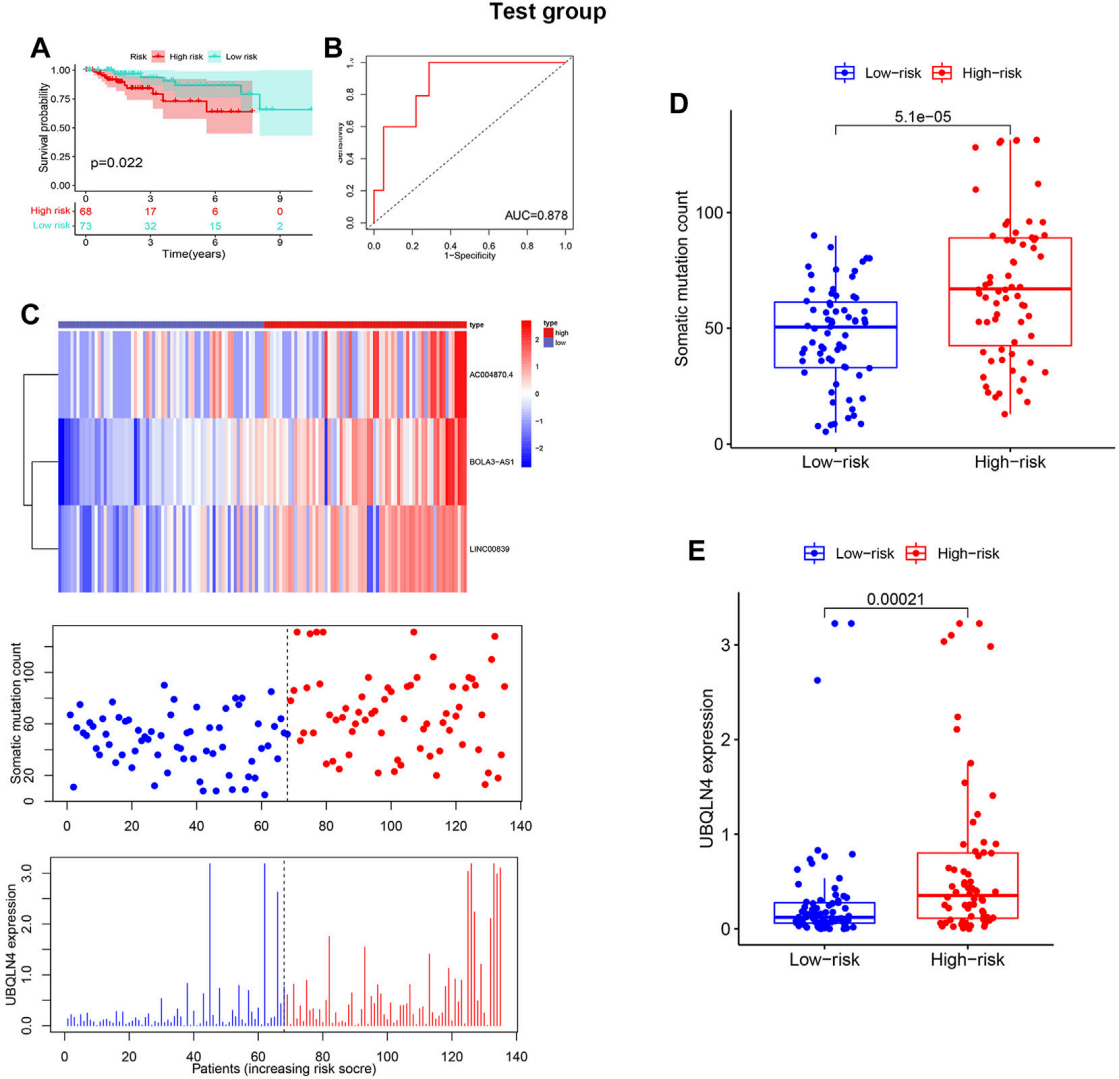
Verification of the GILncSig in the test set. **(A)**. Kaplan–Meier survival curves of GILncSig in the test set. **(B)**. Receiver operating characteristic curves of GILncSig in the test set at 3 years. The distribution trend of somatic mutation and UBQLN4 expression increased in correlation with rising GILncSig in the test set **(C)**. Comparison of overall cell mutation count and UBQLN4 expression level between the risk^hi^ and risk^lo^ groups in the test set **(D,E)**.

**FIGURE 6:**
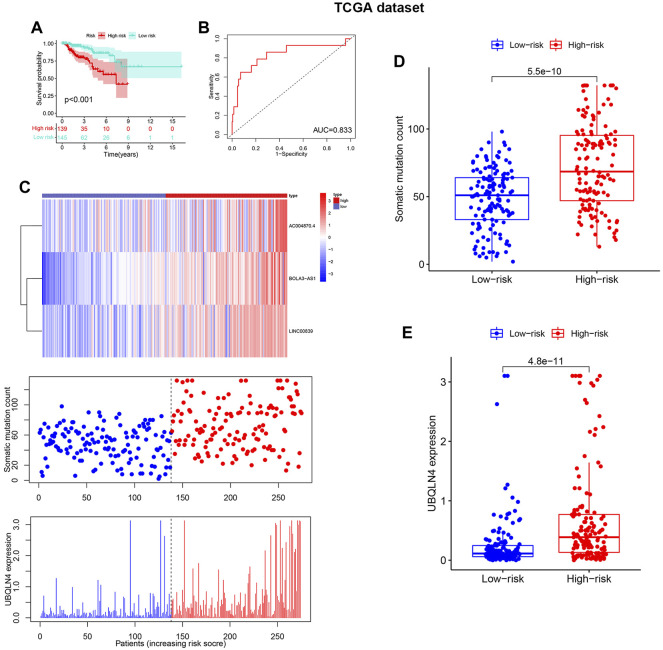
Verification of the GILncSig in the Cancer Genome Atlas (TCGA) datasets. **(A)**. Kaplan–Meier survival curves of GILncSig in the TCGA dataset. **(B)**. Receiver operating characteristic curves of GILncSig at 3 years in the TCGA dataset. **(C)**.The distribution trend of somatic mutation and UBQLN4 expression increased in correlation with rising GILncSig in the TCGA dataset. The number of somatic mutations between the risk^hi^ and risk^lo^ groups and the expression level of UBQLN4 in the risk^hi^ and risk^lo^ groups **(D,E)**.

The test and TCGA sets were used to verify the prognostic value of GILncSig. The test set, which included 142 patients, showed that the survival rate of the high-risk group (*n* = 85) was much lower than that of the risk^lo^ group (*n* = 91) (Figure 5A), with an AUC of 0.878 (Figure 5B). Additionally, the test set showed that the expression of GILncSig and the expression level of UBQLN4, as well as somatic mutation number were similar to the training set (Figure 5C). The test group showed substantial difference in the number of somatic mutations count between the risk^hi^ and risk^lo^ groups (Figure 5D); in addition, UBQLN44 expression in the high-risk group was higher than that in the risk^lo^ group (Figure 5E). Moreover, GILncSig in the TCGA dataset showed similar results. The overall survival rate in the risk^hi^ group was significantly lower than that in the risk^lo^ group (*p* < 0.001, Figure 6A), with an AUC of 0.833 for the TCGA set (Figure 6B). The TCGA set also showed similar GILncSig expression, somatic mutation count and UBQLN4 expression (Figure 6C). In the TCGA dataset, higher somatic mutation numbers were observed in the high-risk group (Figure 6D). It was revealed that there was a statistically significant difference in UBQLN4 expression between the two risk groups (Figure 6E).

### GILncSig is Prognostically Independent of Other Clinicopathological Factors

Univariate Cox and multivariate Cox analysis on the age, gender, pathological stage malignancy and prognostic risk score of GILncSig was performed. GILncSig was found to be an independent prognostic predictor in patients with KIRP after controlling for age, gender, and clinical stage (Supplementary Figure S2A,B).

### GILncSig’s Performance is Compared to Those of Other lncRNA-Related Signatures and Molecular Biomarkers

**FIGURE 7:**
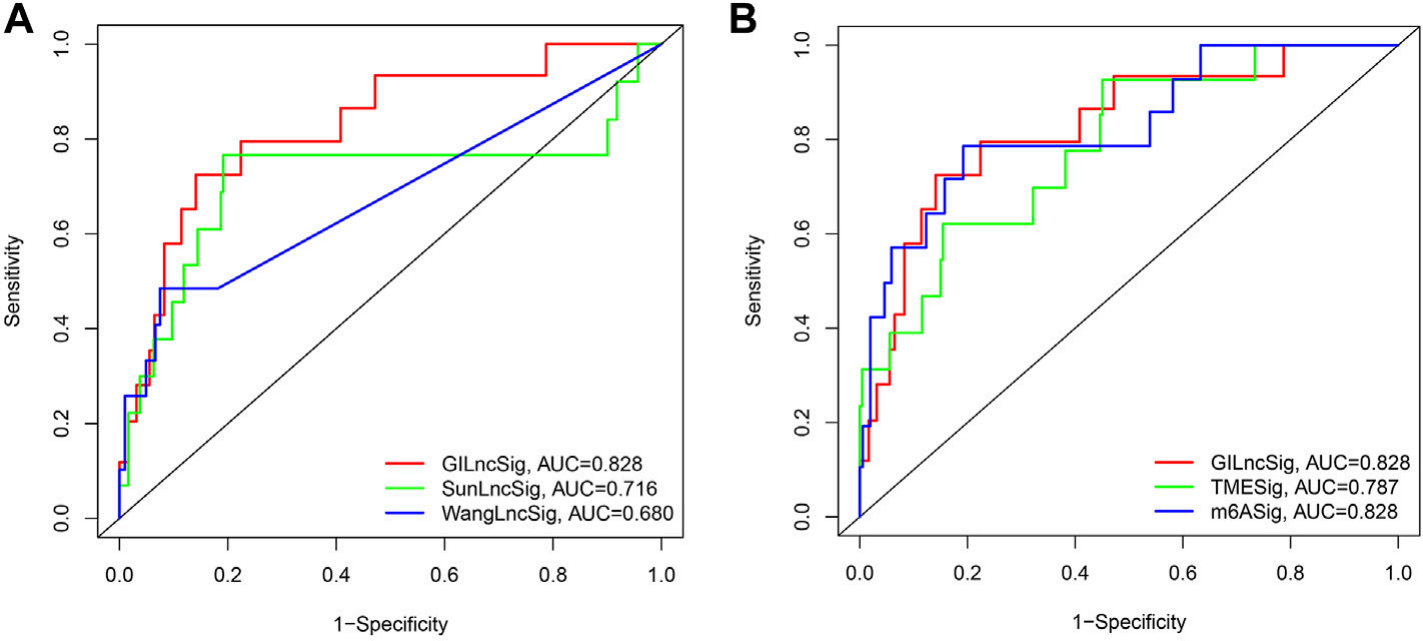
Receiver operating characteristic analyses of the GILncSig, other lncRNA-related signatures and molecular biomarkers. **(A)** Receiver operating characteristic analyses of the GILncSig, WanglncSig and SunlncSig at 3 years. **(B)** Receiver operating characteristic analyses of the GILncSig, m6ASig and TMESig at 3 years.

The predictive performance of GILncSig was compared with two recently published lncRNA characteristics. A 3-lncRNA signature was obtained from a study conducted by Wang (LilncSig is the name given to this signature), and an 8-lncRNA signature was obtained from a work conducted by Sun (referred to as SunlncSig). The AUC of the 3-year overall survival (AUC = 0.828) of GILncSig was higher than that of Wanglncsig (AUC = 0.680) and Sunlncsig (AUC = 0.716) (Figure 7A), indicating that GILncSig has a higher predictability when compared to the other two previously reported lncRNA signatures. Besides, the predictive value of GILncSig was further compared with two KIRP related molecular biomarkers. A 3-m6A signature (m6ASig) was built form a research by Wang (Sun Z. et al., 2020), and a 4 tumor microenvironment signature (TMESig) was constructed form a study by Su (Luo et al., 2021). The results showed that the AUC of the 3-year overall survival of GILncSig was higher than that of TMESig (AUC = 0.787), in addition, it has the same value as m6ASig (AUC = 0.828) (Figure 7B).These data suggest that GILncSig is a promising biomarker in KIRP.

## Discussion

KIRP remains a clinical challenge owing to its high histological heterogeneity, poor prognosis and limited treatment options. The majority of the research on KIRP has been on a few well-known cancer-related genes, for example, hsa-mir-3199 and hsa-mir-1293 were found to be novel prognostic biomarkers of KIRP (Luo et al., 2017). Additionally, several mRNAs were discovered to be predictive of the survival probability of KIRP patients (Gao et al., 2019), such as CCNB2, IGF2BP3, KIF18A, PTTG1, and BUB1. However, the incidence and mortality rates of KIRP have been annually increasing; however, the underlying mechanism of its carcinogenesis remains unclear. Therefore, identifying new reliable molecular features to predict the survival rate of patients with KIRP is crucial. Technologies such as high-throughput and transcriptome sequencing have largely promoted cancer research in the past decade. This study discovered a new signature based on three lncRNAs that are related with genomic instability that can be used to predict the prognosis of patients with KIRP, which was previously unknown, as well as the prognostic valve of these signature was confirmed using data from TCGA. The prognostic signature developed exhibited good performance in distinguishing high-risk patients with KIRP that had a poor prognosis, which may contribute to their clinical treatment.

lncRNA, an important noncoding RNA, is a critical player in cell homeostasis, cell proliferation, migration, and genome stability, among other processes (Huarte, 2015). The lncRNA GClnc1 is linked to the malignant behaviour of bladder cancer (Zhuang et al., 2019). Previous research has found a number of long noncoding RNAs (lncRNAs) that are linked with the progression and prognosis of RCC. For instance, HCP5 inhibits the growth and metastasis of RCC cells by regulating the mir-214-3p/mapk1 axis (Hao et al., 2020). Moreover, lncRNAs are closely associated with the incidence of RCC and have been identified as a new prognostic marker in recent years (Bhan et al., 2017; Wang et al., 2019). For example, Xia et al. developed a result prediction model based on nine lncRNAs with redox-related functions (Qi-Dong et al., 2020). Additionally, Zhang et al. identified five lncRNAs associated with the overall survival of RCC (Zhang et al., 2021). LncRNAs, in addition, are strongly associated with genomic stability and have been shown to enhance the onset, progression, and metastasis of RCC (Lee et al., 2016; Zhai et al., 2017). A recent study reported six genomic instability-associated lncRNAs (LINC02678, HOXA10-AS, RHOXF1-AS1, AC010789.1, LINC01150, and TGFB2-AS1) that were associated with gastric cancer prognosis and somatic mutations (Sun et al., 2021). However, studies reporting on lncRNAs associated with genomic instability in KIRP are limited.

3,840 cancer-associated lncRNAs were retrieved from the TCGA database and 27 lncRNAs related to genomic instability were found in this study to further investigate the potential impacts of genomic instability on KIRP. Cox regression analysis showed that 11 genomic instability-associated lncRNAs were related to the survival of patients with KIRP. Furthermore, The GILncSIg was constructed using three mutation-derived lncRNAs, which can be used to predict the outcome of KIRP patients. Among these lncRNAs with predictive characteristics related to genome instability, BOLA3-AS1, LINC00839, and AC004870.4 were considered the risk factors. BOLA3-AS1 is a different transcript of BOLA3 (Fagerberg et al., 2014), which is associated with high-risk myelodysplastic syndrome and plays a crucial function in the formation of blood cells, such as platelets, erythrocytes and bone marrow cells (Szikszai et al., 2020). Furthermore, the expression of BOLA3-AS1 has been reported to be up-regulated in gastric cancer cell lines and is related to the poor prognosis of gastric cancer (Zhang et al., 2021). Studies have shown that LINC00839 targets the mir-338-3p/GLUT1 axis to promote the cell proliferation, migration, invasion and glycolysis of neuroblastoma cells, resulting in the poor survival rate of patients with neuroblastoma (Sahu et al., 2018; Yang et al., 2021). In addition, LINC00389 promotes the proliferation, migration, and invasion and inhibits the apoptosis of liver and lung cancer cells (Meng et al., 2021; Yu et al., 2021). However, as far as we are aware at this time, AC004870.4 has been published for the first time in this study. Therefore, well-designed investigations should be carried out in the future to explore potential functions and mechanisms of AC004870.4 in cancer, which may reveal their potential as therapeutic targets for patients with KIRP.

Furthermore, we analysed the characteristics of GILncSIg based on the clinical information of KIRP. The survival curve revealed that patients in the high-risk group had a lower overall survival rate than those in the low-risk group. Notably, the results of time-dependent ROC curve analysis showed that the AUC value of GILncSig in the training and test sets exceeded 0.60, indicating the accuracy of the signature. These results were verified using the GEO dataset, which showed similar results. Moreover, the time-dependent ROC curve revealed that the signature developed in this study is an independent prognostic factor when compared with other clinicopathological features (including age, sex, and tumour stage). Other prognostic features of KIRP have been previously reported. However, in terms of ROC analysis, the signature established in this study has been proven to be more accurate in predicting survival outcomes than the signatures reported in previous studies. Furthermore, we assessed whether genomic instability was related to GILncSIg and found that the tumour mutation phenotype and UBEQL4 expression level were important indicators of genomic instability. UBEQL4 is a novel regulator of cancer genomic instability, which can lead to genomic instability to inhibit homologous recombination-mediated DSB repair (HRR) activity (Jachimowicz et al., 2019). The results revealed that the somatic mutation rate and expression level of UBEQL4 in the high-risk group were significantly higher than those in the low-risk group. This finding indicated that GILncSIg was closely related to genomic instability and further demonstrated the key role of lncRNAs in maintaining genomic instability and the importance of examining the underlying mechanisms of lncRNAs in epigenetics. Although this study provides promising insights into a better genomic instability assessment and KIRP prognosis, it had some limitations. First, although GILncSig was verified using the TCGA and GEO datasets, it will be necessary to use more independent datasets in order to further verify GILncSig in order to confirm its robustness and reproducibility. Second, it is necessary to conduct biological experiments on these predicted lncRNAs *in vitro* and *in vivo*. In conclusion, this study proposes a computational framework derived from a hypothesis based on mutations to identify lncRNAs related to genomic instability, which introduces novel outlooks and resources for further studying the role of lncRNAs in genomic instability. Based on lncRNA expression profile, somatic mutation profile and clinical data of KIRP patients, a prognostic marker of lncRNA derived from genomic instability was constructed to predict the prognosis of people suffering from KIRP. GILncSig was found to be a potential prognostic index that was independent of other usual clinicopathological factors, according to further investigation, thereby introducing a new target for KIRP treatment.

## Data Availability

The datasets presented in this study can be found in online repositories. The names of the repository/repositories and accession number(s) can be found in the article/Supplementary Materials.
