# Supplementary material for: Comprehensive Analyses of Mutation-Derived Long-Chain Noncoding RNA Signatures of Genome Instability in Kidney Renal Papillary Cell Carcinoma
Source: Front Genet. 2022 Apr 25;13:874673. doi: 10.3389/fgene.2022.874673 (PMC9082950; doi:10.3389/fgene.2022.874673)
Supplement: Supplementary file 1 [file DataSheet2.PDF]

SUPPLEMENTARY TABLE 2. Multivariate cox regression analysis of the long-chain noncoding RNAs (lncRNAs) related to prognosis in patients with kidney renal papillary cell carcinoma.

id	coef	HR	HR.95L	HR.95H	*p* -value
`BOLA3-AS1`	0.734368853	2.084166162	0.904038849	4.804825143	0.008484574
AC004870.4	0.688625061	1.990976179	1.247511268	3.177515304	0.003887188
LINC00839	0.33935639	1.404043644	0.942733121	2.091088675	0.009496019
