# Supplementary figures and images for: Comprehensive Analyses of Mutation-Derived Long-Chain Noncoding RNA Signatures of Genome Instability in Kidney Renal Papillary Cell Carcinoma
Source: Front Genet. 2022 Apr 25;13:874673. doi: 10.3389/fgene.2022.874673 (PMC9082950; doi:10.3389/fgene.2022.874673)

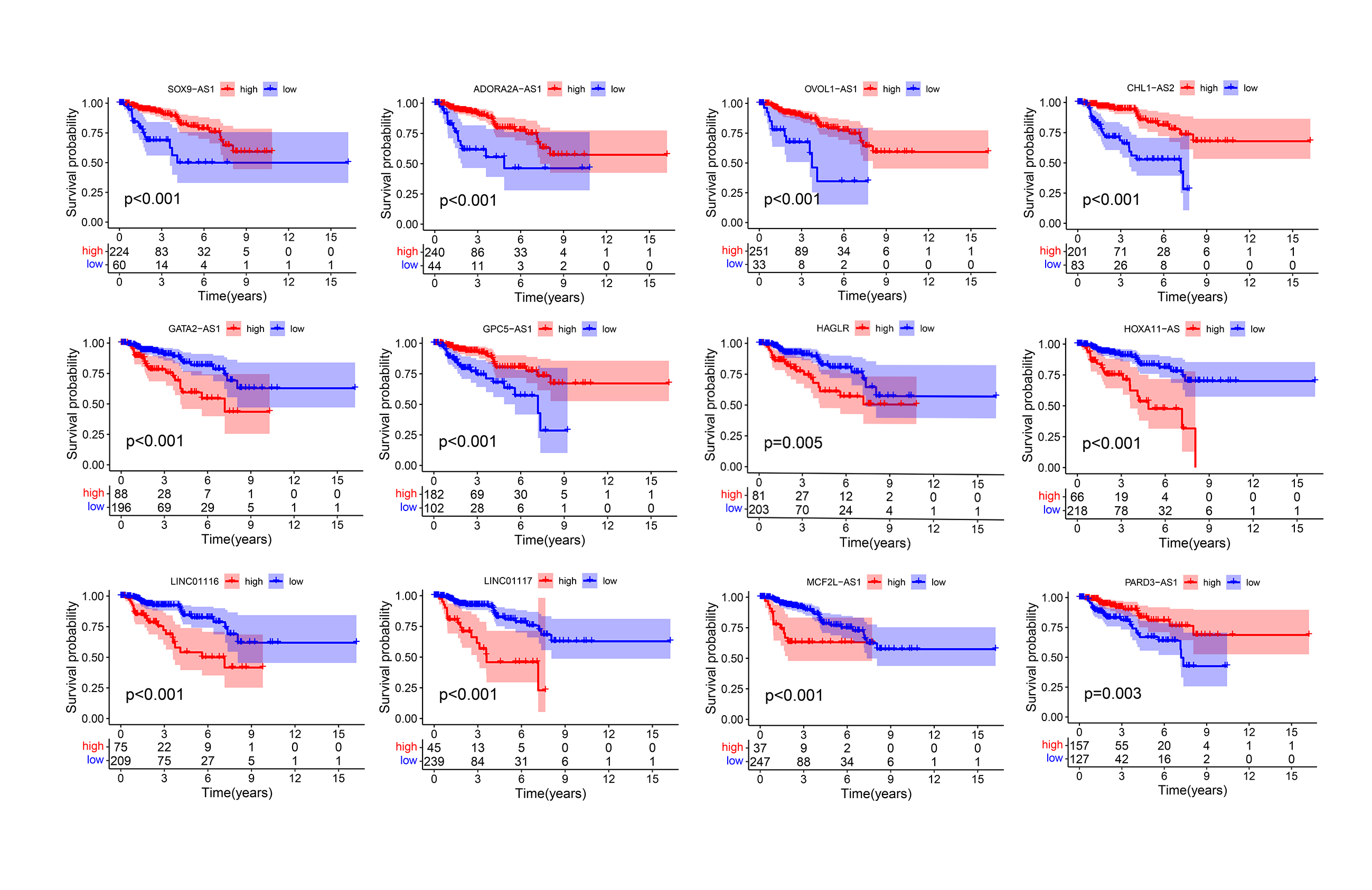

Supplement: Supplementary file 2 [file Image1.JPEG]

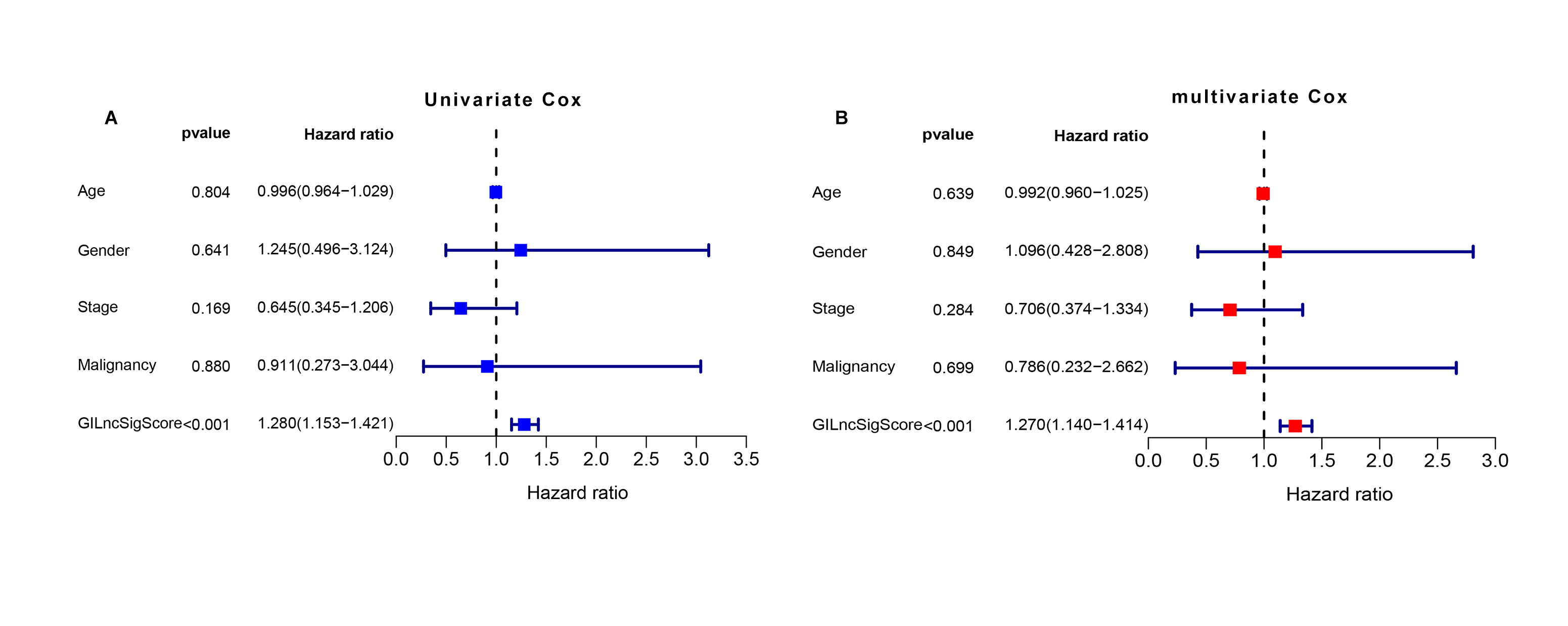

Supplement: Supplementary file 3 [file Image2.JPEG]
