# Supplementary material for: Comprehensive Analyses of Mutation-Derived Long-Chain Noncoding RNA Signatures of Genome Instability in Kidney Renal Papillary Cell Carcinoma
Source: Front Genet. 2022 Apr 25;13:874673. doi: 10.3389/fgene.2022.874673 (PMC9082950; doi:10.3389/fgene.2022.874673)
Supplement: Supplementary file 4 [file DataSheet1.PDF]

SUPPLEMENTARY TABLE 1. List of the 27 differentially expressed long-chain noncoding RNAs (lncRNAs) between the genomic stable (GS) and genomic unstable (GU) subgroups

id	conMean	treatMean	log2FC	*p* -Value
MCF2L-AS1	0.863225507	0.075607058	-3.513144678	1.22E-05
BOLA3-AS1	1.298352333	0.850134166	-0.610919493	2.05E-05
HOXA11-AS	0.801660879	0.300775247	-1.414306228	0.006727994
XIST	1.027610803	0.505315719	-1.024036998	0.002172049
GATA2-AS1	0.718460743	0.25171818	-1.513099914	0.000319
OVOL1-AS1	0.623513002	1.177855096	0.917670516	1.33E-05
LINC00543	0.491754715	0.797484342	0.697517307	7.91E-05
LINC01460	0.360836528	0.571029138	0.662218973	0.003610249
SMIM2-AS1	2.023355396	3.072046655	0.602450379	4.92E-08
PARD3-AS1	1.482146287	2.317769847	0.645049467	7.72E-07
TMEM246-AS1	0.448508175	0.763536193	0.767562263	0.000672664
LAMA5-AS1	0.367412443	0.560983775	0.610558559	0.002155516
CHL1-AS2	1.403396926	2.317867466	0.723874969	0.000191223
LINC01117	0.807174674	0.529333802	-0.608703125	0.040879729
HAGLROS	1.016212068	0.655289862	-0.632996385	0.003501232
AL356310.1	0.267546463	0.603287504	1.173056242	0.002721933
MIR4500HG	0.315564236	0.541393867	0.778744834	0.002578869
SOX9-AS1	0.541391242	0.814726284	0.589643902	0.000140544
HAGLR	2.074427398	1.380207164	-0.58782834	0.000473285
ADORA2A-AS1	0.380277394	0.665483437	0.80735058	0.000230234
HOXB-AS3	1.534807893	1.00554093	-0.610086283	0.009539209
GPC5-AS1	0.57825801	1.009123969	0.803318166	0.018135679
LINC00671	0.92825743	1.69035326	0.864727919	0.000120461
AC004870.4	0.711121978	0.152087022	-2.225199997	0.031564048
LINC01116	2.115023486	1.384179011	-0.61164315	0.003158905
LINC00839	1.233070716	0.518799729	-1.249005908	0.000418986
AF131215.2	0.972364015	0.642314273	-0.598217148	0.001338234
